# Pharmacological treatment for patients with obesity and heart failure: Focus on glucagon‐like peptide‐1 receptor agonists. *European Journal of Heart Failure* expert consensus document

**DOI:** 10.1002/ejhf.70082

**Published:** 2025-11-27

**Authors:** Luca Monzo, Gianluigi Savarese, Wilfried Mullens, Amr Abdin, Biykem Bozkurt, Ovidiu Chioncel, Seif El Hadidi, Thomas M. Gorter, Riccardo M. Inciardi, Mark C. Petrie, Gabriele G. Schiattarella, Davide Stolfo, Marco Metra, Nicolas Girerd

**Affiliations:** ^1^ Centre d'Investigations Cliniques Plurithématique 1433 and INSERM U1116 ‐ DCAC, CHRU Nancy, F‐CRIN INI‐CRCT (Cardiovascular and Renal Clinical Trialists) Université de Lorraine Nancy France; ^2^ Department of Clinical Science and Education, Södersjukhuset; Karolinska Institutet Stockholm Sweden; ^3^ Department of Cardiology, Ziekenhuis Oost‐Limburg A.V. Genk Belgium; ^4^ Hasselt University, Faculty of Medicine and Life Sciences Diepenbeek Belgium; ^5^ Internal Medicine Clinic III, Cardiology, Angiology and Intensive Care Medicine Saarland University Hospital Homburg Germany; ^6^ Winters Center for Heart Failure Research, Cardiovascular Research Institute Baylor College of Medicine Houston TX USA; ^7^ Emergency Institute for Cardiovascular Diseases ‘Prof. C.C. Iliescu’ Bucharest Romania; ^8^ University of Medicine Carol Davila Bucharest Romania; ^9^ Royal College of Surgeons in Ireland (RCSI) Dublin Ireland; ^10^ Department of Cardiology, University of Groningen University Medical Centre Groningen Groningen The Netherlands; ^11^ Institute of Cardiology, ASST Spedali Civili di Brescia and Department of Medical and Surgical Specialties, Radiologic Sciences, and Public Health University of Brescia Brescia Italy; ^12^ School of Cardiovascular and Metabolic Health University of Glasgow Glasgow UK; ^13^ Deutsches Herzzentrum der Charité, Department of Cardiology, Angiology and Intensive Care Medicine, Max Rubner Center for Cardiovascular Metabolic Renal Research (MRC) Berlin Germany; ^14^ DZHK (German Centre for Cardiovascular Research), Partner Site Berlin Berlin Germany; ^15^ Translational Approaches in Heart Failure and Cardiometabolic Disease, Max Delbrück Center for Molecular Medicine in the Helmholtz Association (MDC) Berlin Germany; ^16^ Friede Springer Cardiovascular Prevention Center at Charité – Universitätsmedizin Berlin Berlin Germany; ^17^ Experimental and Clinical Research Center (ECRC), a Cooperation of Charité‐Universitätsmedizin Berlin and Max Delbruck Center for Molecular Medicine (MDC) Berlin Germany; ^18^ Division of Cardiology, Department of Advanced Biomedical Sciences Federico II University Naples Italy; ^19^ Cardiology, Cardiothoracic Department, Azienda Sanitaria Universitaria Friuli Centrale (ASUFC) Udine Italy

**Keywords:** Heart failure, Glucagon‐like peptide‐1 receptor agonists, Obesity, HFpEF

## Abstract

There is growing clinical interest in strategies for improving clinical outcomes in patients with heart failure (HF) and obesity. The development of glucagon‐like peptide‐1 receptor agonists (GLP‐1 RAs) and of the dual glucose‐dependent insulinotropic polypeptide (GIP)/GLP‐1 RA has expanded therapeutic options for this population. This expert consensus provides a comprehensive and pragmatic framework for the use of GLP‐1 RAs and GIP/GLP‐1 RA in patients with HF, focusing on clinical integration, patient selection, safety, and tolerability. We review the evidence supporting their use in patients with HF with preserved ejection fraction (HFpEF), where clinical trials have demonstrated meaningful reductions in body weight alongside improvements in health status and exercise capacity. Whether these effects translate into fewer HF events or lower cardiovascular mortality remains uncertain, as current evidence is limited to two small trials with few observed events. In contrast, data regarding the efficacy and safety of these drugs in HF with reduced ejection fraction are scarce, with dedicated outcome trials yet to be launched. Finally, this document highlights knowledge gaps and outlines future research directions in this field.

## Introduction

The clinical characteristics of patients with heart failure (HF) have radically changed in the last decades with a decrease in coronary artery disease as a cause of HF with reduced ejection fraction (HFrEF) and a rise in HF with preserved ejection fraction (HFpEF), caused by metabolic disorders and hypertension. Specific characteristics of patients with obesity and HFpEF have also been outlined and an obese phenotype of HFpEF is now described.^1^, ^2^, ^3^ Notably, in obese HF patients circulating natriuretic peptide levels are often lower than expected for the degree of haemodynamic congestion, complicating diagnosis and risk stratification.^4^

Advances in our understanding of the gut–brain axis in appetite and metabolic regulation have paved the way for novel therapeutic approaches.^5^ Central to this axis are nutrient‐stimulated enteroendocrine hormones, such as glucagon‐like peptide‐1 (GLP‐1) and glucose‐dependent insulinotropic polypeptide (GIP),^5^ which are now pharmacologically targeted by a new generation of agents that mimic or amplify their physiological actions. GLP‐1 is secreted by intestinal L‐cells in response to nutrient ingestion, and plays a key role in postprandial glucose homeostasis by enhancing glucose‐dependent insulin secretion, suppressing glucagon release, delaying gastric emptying, and promoting satiety.^6^ Moreover, the expression of GLP‐1 receptors in cardiac tissue suggests direct myocardial effects, such as enhanced cardiomyocyte survival, improved cardiac glucose utilization, and reduced ischemia–reperfusion injury.^7^ In contrast, GIP receptors are predominantly expressed in adipose tissue, where they contribute to lipid handling by activating lipoprotein lipase.^8^

The advent of GLP‐1 receptor agonists (GLP‐1 RAs) and the dual GIP/GLP‐1 RA tirzepatide marks a paradigm shift in the treatment of obesity and the obese phenotype of HFpEF.^9^ These agents promote weight loss, primarily through appetite suppression and reduced energy intake, and improve glycaemic control in a glucose‐dependent manner, thereby minimizing the risk of hypoglycaemia. Notably, some GLP‐1 RAs (i.e. semaglutide, liraglutide) cross the blood–brain barrier and act on hypothalamic centres to enhance satiety, leading to reduced food intake and preferential loss of fat mass.^10^

No specific guidance exists for the use of these agents in patients with obesity and HF. This *European Journal of Heart Failure* expert consensus seeks to fill that gap by integrating available evidence with the collective appraisal of field experts, providing a comprehensive framework for the clinical integration of GLP‐1 RAs, while also exploring key controversies and offering insights on therapeutic positioning within the broader context of HF management.

## Overview of existing evidence in heart failure

### Potential weight‐independent benefits of GLP‐1 RAs

While no mechanistic trials have specifically examined GLP‐1 RAs in HF, emerging data suggest potential benefits beyond weight reduction, possibly mediated by the widespread distribution of GLP‐1 receptors across multiple organ systems.^9^ In both STEP‐HFpEF (Semaglutide Treatment Effect in People with Obesity and Heart Failure with Preserved Ejection Fraction) and SUMMIT (Tirzepatide for Heart Failure with Preserved Ejection Fraction and Obesity) trials, reductions in inflammatory markers (i.e. C‐reactive protein) and in N‐terminal pro‐B‐type natriuretic peptide (NT‐proBNP) were observed before the peak of weight loss,^11^, ^12^, ^13^ contrasting with prior trials of lifestyle and dietary interventions where natriuretic peptides often increased.^14^ In these trials, improvements in health status and 6‐min walk distance correlated with the magnitude of weight loss. However, even participants who lost <5% of their body weight achieved clinically meaningful gains in Kansas City Cardiomyopathy Questionnaire (KCCQ; mean increases of 5.3 points in the overall score), exceeding those typically observed in prior trials of sacubitril/valsartan, spironolactone, or sodium–glucose co‐transporter 2 (SGLT2) inhibitors, where mean KCCQ improvements ranged from 0.5 to 3.0 points.^15^, ^16^, ^17^ In multivariable regression, only ~20–30% of the treatment effect on symptoms and exercise capacity was attributable to weight loss alone.^15^ Tirzepatide also showed a potential benefit on proinflammatory paracardiac fat.^18^ In the cardiac magnetic resonance substudy of the SUMMIT trial, treatment reduced pericardial adipose tissue volume and left ventricular mass compared with placebo, but did not affect epicardial adipose tissue.^19^ Further analyses showed that tirzepatide reduced estimated blood volume, C‐reactive protein, NT‐proBNP level, and troponin T levels compared with placebo.^20^

Potential weight loss‐independent benefits have also been suggested by observations in other populations. In the Harmony Outcomes (Albiglutide and cardiovascular outcomes in patients with type 2 diabetes and cardiovascular disease) trial, albiglutide reduced HF hospitalizations by 20% despite modest effects on weight.^21^ Similarly, reductions in HF events have been observed in cardiovascular (CV) outcome trials of GLP‐1 RAs at non‐weight loss doses, including in patients without obesity.^22^ In the FLOW (Evaluate Renal Function with Semaglutide Once Weekly) trial, semaglutide reduced HF events in a predominantly non‐obese population with diabetic chronic kidney disease.^23^ In the SELECT trial (Semaglutide Effects on Heart Disease and Stroke in Patients With Overweight or Obesity), semaglutide reduced the composite of CV death, non‐fatal myocardial infarction, or non‐fatal stroke by 20% over 5 years in adults with overweight or obesity, independent of the degree of weight loss.^24^ Benefits were also observed in participants without obesity (body mass index [BMI] <30 kg/m^2^) and in those losing <5% of their body weight.^25^ Altogether, these findings suggest a possible role of GLP‐1 RAs in modulating HF outcomes perhaps through mechanisms not entirely dependent on body weight reduction, such as improvements in endothelial function, reductions in oxidative stress and inflammation, myocardial substrate utilization, and modulation of natriuresis, that contribute to CV and renal protection. However, given the challenges in disentangling mediation pathways in treatment effects, the proportion of benefit that is truly independent of weight loss remains uncertain. The magnitude and even the existence of such hypothesized non‐weight loss mechanisms will need to be confirmed in dedicated mechanistic trials in patients with HF.

#### GLP‐1 RAs in HFpEF

A high proportion of patients with HFpEF are obese and an even higher proportion has adiposity.^26^ Obesity is also associated with specific mechanisms and clinical characteristics in patients with HFpEF (so‐called obese phenotype of HFpEF).^1^, ^2^, ^3^ Of note, an ‘obesity paradox’ has been reported in HF, with observational studies suggesting lower mortality among patients with higher BMI. However, this finding remains controversial and is likely explained by confounding factors,^27^ as more recent analyses using alternative metrics of adiposity (i.e. waist‐to‐hip ratio) have suggested the opposite.^28^ Consistently, intentional weight loss achieved through bariatric surgery has been associated with a reduced long‐term risk of incident HF and CV events,^29^ supporting the potential benefits of weight reduction when accomplished in a controlled setting.

Evidence supporting the use of GLP‐1 RAs and dual GIP/GLP‐1 RA is more consistent in patients with HFpEF and obesity. In this setting, semaglutide has shown benefits beyond weight loss, including improvements in quality of life, functional status, and exercise capacity. In the STEP‐HFpEF trial (*n* = 529, LVEF ≥45%, median BMI 37 kg/m^2^), semaglutide 2.4 mg once weekly led to greater reductions in body weight (−10.7%), improvements in KCCQ (+7.8 points), increased 6‐min walk distance (+20.3 m), and lower C‐reactive protein and NT‐proBNP levels compared to placebo at 52 weeks, in patients without type 2 diabetes mellitus (T2DM), regardless of baseline BMI, left ventricular ejection fraction (LVEF) and New York Heart Association (NYHA) class.^11^, ^30^ A benefit was shown on a hierarchical composite endpoint (death, HF events, and differences in the change in the KCCQ and 6‐min walk distance). Similar findings were reported in the STEP‐HFpEF DM trial (*n* = 616), which included patients with obesity, HFpEF, and diabetes.^12^ However, with only 38 HF events across the two STEP‐HFpEF trials, conclusions regarding effects on clinical outcomes remain limited despite apparently favourable signals. A pooled patient‐level meta‐analysis of 3743 patients from the STEP‐HFpEF, STEP‐HFpEF DM, SELECT, and FLOW trials reported that semaglutide reduced the risk of CV death or worsening HF by 31% (103 [5.4%] of 1914 in the semaglutide group had events vs. 138 [7.5%] of 1829 in the placebo group; hazard ratio [HR] 0.69, 95% confidence interval [CI] 0.53–0.89]; *p* = 0·0045), largely driven by fewer HF events (54 [2.8%] vs. 86 [4.7%]; HR 0.59, 95% CI 0.41–0.82; *p* = 0·0019), with a more modest effect on CV death (59 [3.1%] vs. 67 [3.7%]; HR 0.82, 95% CI 0.57–1.16; *p* = 0·25).^31^ However, the limited number of events and lack of detailed HF phenotyping in SELECT and FLOW temper the strength of these conclusions.

Recently, the SUMMIT trial assessed the dual GIP/GLP‐1 RA tirzepatide (subcutaneous, once‐weekly, up to 15 mg) in 731 patients with obesity‐related HFpEF (LVEF ≥50%; median BMI 38 kg/m^2^). Similar to STEP‐HFpEF, tirzepatide resulted in significant improvements in HF‐related symptoms, exercise capacity, and weight loss. Moreover, it demonstrated a reduction in the risk of the composite endpoint of CV death or worsening HF compared to placebo, although with a very small number of events (56 events [15.3%], 8.8 events per 100 patient‐years on placebo vs. 36 events [9.9%], 5.5 events per 100 patient‐years with tirzepatide).^13^ Specifically, only 38 of these events were HF hospitalizations, further limiting the ability to draw firm conclusions about a potential reduction in HF events. A meta‐analysis of six randomized controlled trials (RCTs) involving 8788 patients showed a significant reduction in the composite outcome of CV death or worsening HF events (HR 0.68, 95% CI 0.51–0.89; *p* = 0.006), as well as of worsening HF events alone (HR 0.56, 95% CI 0.38–0.82; *p* = 0.003).^32^ Nonetheless, also this analysis remains constrained by the limited number of events.

However, while STEP‐HFpEF and SUMMIT provide important signals of benefit in patients with obesity‐related HFpEF, several limitations of these pivotal trials warrant caution in interpreting the results. Both showed notable discontinuation rates (semaglutide: 13.3%; tirzepatide: 6.3%), mainly due to gastrointestinal side effects, and a quite high overall dropout (STEP‐HFpEF: 16% in both arms; SUMMIT: ~20% in both arms), which may introduce bias. Most participants were in NYHA class II (~70%), while those with BMI <30 kg/m^2^ were excluded, even though many individuals in this category may have excess visceral adiposity, limiting generalizability of the results to this population. Both trials demonstrated substantial improvements in health status as measured by the KCCQ, with a magnitude of benefit far exceeding that observed in prior HF therapy trials. Nevertheless, given the marked weight loss differences between active treatment and placebo, blinding may not have been fully maintained, and subjective measures should therefore be interpreted with caution. In SUMMIT, median NT‐proBNP levels were relatively low (median 196 pg/ml), raising concerns about diagnostic robustness, particularly as nearly half of the patients had a recent HF hospitalization but very few events occurred thereafter, including in the control arm.

In summary, while semaglutide and tirzepatide show promise for obesity‐related HFpEF, further validation is needed to confirm their safety, long‐term efficacy, and role alongside lifestyle interventions. Whether weight loss‐associated benefits translate into a reduction in clinical events awaits confirmation in large, event‐driven outcome trials across diverse populations.

#### GLP‐1 RAs in HFrEF

Glucagon‐like peptide‐1 receptor agonists have been specifically evaluated in patients with HFrEF in small RCTs. In the LIVE (Effect of Liraglutide on Left Ventricular Function in Stable Chronic Heart Failure Patients With and Without Diabetes) trial, 241 patients (median BMI 29 kg/m^2^ – so less than 50% were obese) with stable chronic HFrEF (LVEF ≤45%), with or without T2DM, were randomized to liraglutide (1.8 mg daily – i.e. not a weight loss dose) or placebo. After 24 weeks, liraglutide showed no effect on LVEF, quality of life, or symptoms, but was associated with a higher incidence of serious cardiac adverse events (10.0% vs. 3.0%, *p* = 0.04). Importantly there was only one death and one HF hospitalization in the ‘serious adverse event’ composite endpoint.^33^ The FIGHT (Functional Impact of GLP‐1 for Heart Failure Treatment) trial enrolled 300 HFrEF patients who had been hospitalized within 2 weeks (median BMI 32 kg/m^2^) and found no benefit of a non‐weight loss dose of liraglutide (1.8 mg/day) on the primary hierarchical endpoint of death, HF hospitalization, and change in NT‐proBNP.^34^ Patients had significant disease burden, with 60% in NYHA class III, 88% with a recent hospitalization, median LVEF 25%, median NT‐proBNP 2000 pg/ml, and median creatinine 1.5 mg/dl. A post‐hoc analysis suggested potential harm in NYHA class III–IV patients, with increased risk of arrhythmias and worsening HF.^35^ In a pooled analysis of FIGHT and EXSCEL (Exenatide Study of Cardiovascular Event Lowering) trial, which studied exenatide in T2DM patients with or without CV disease, GLP‐1 RA use was associated with a higher risk of HF hospitalization in patients with LVEF <40% (odds ratio [OR] 1.49, 95% CI 1.05–2.10).^36^ Similarly, a meta‐analysis including SELECT, FLOW, and EXSCEL suggested that GLP‐1 RAs may increase the risk of worsening HF events in patients with HFrEF.^37^

In contrast, a prespecified analysis of the SELECT trial reported that semaglutide (2.4 mg daily) reduced the risk of the primary composite endpoint (CV death, non‐fatal myocardial infarction, or non‐fatal stroke) in patients with atherosclerotic CV disease and overweight or obesity (BMI ≥27 kg/m^2^), with and without HF and regardless of HF subtype.^38^ However, several limitations temper the interpretation of these findings in the HF setting. Patients in NYHA class IV were excluded, and over 90% of those with HFrEF were in NYHA class I or II. Moreover, HF classification relied on investigator report without standardized assessment of LVEF, natriuretic peptides, or prior HF hospitalizations, and background HF therapy was incompletely characterized. Additionally, the event rates for HF hospitalization or CV death in the HFrEF subgroup were substantially lower than in contemporary HFrEF trials,^39^, ^40^ suggesting this was a low‐risk population. To date, no large scale RCTs have evaluated GLP‐1RAs or the dual GIP/GLP‐1 RA tirzepatide in HFrEF (with or without obesity).

Overall, based on current evidence, both the safety and efficacy of GLP‐1 RAs and dual GIP/GLP‐1 RAs in patients with HFrEF remain uncertain and require confirmation in dedicated, adequately powered trials across the full spectrum of disease severity.

## Expert advice on the use of GLP‐1 RAs in heart failure

### Patient selection

**Figure 1:**
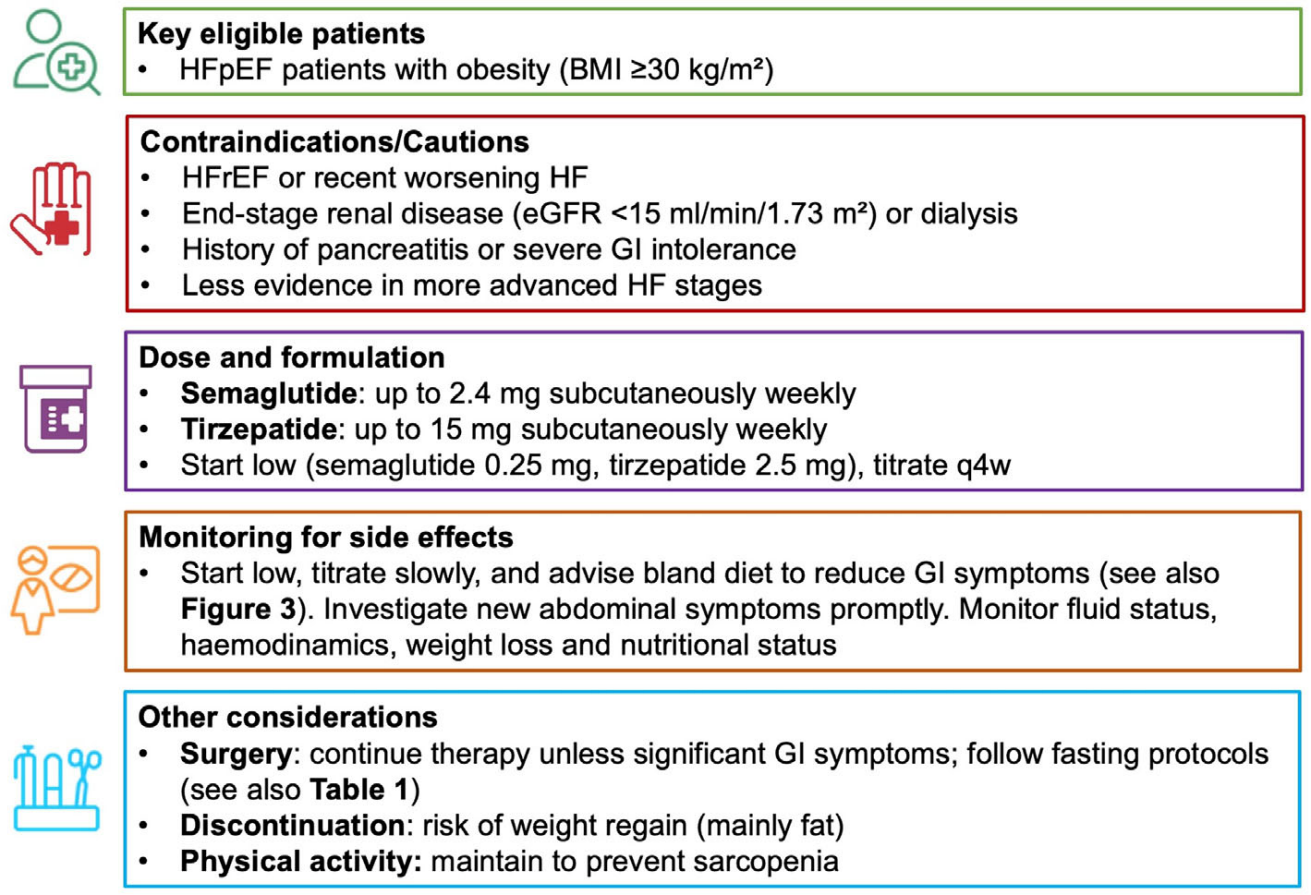
Practical use of glucagon‐like peptide‐1 receptor agonists in patients with heart failure. BMI, body mass index; eGFR, estimated glomerular filtration rate; GI, gastrointestinal; HF, heart failure; HFpEF, heart failure with preserved ejection fraction; HFrEF, heart failure with reduced ejection fraction; q4w, every 4 weeks.

The integration of GLP‐1 RAs and tirzepatide into the therapeutic strategy for patients with HF and obesity requires careful patient selection based on clinical characteristics, HF stage and phenotype (*Figure* *1*).

#### Heart failure phenotype

Evidence to date supports the use of these agents primarily in individuals with HFpEF and obesity (BMI ≥30 kg/m^2^), where semaglutide and tirzepatide have shown consistent benefits in exercise tolerance and quality of life.^11^, ^12^, ^13^, ^38^ By contrast, their role in HFrEF remains uncertain, as small studies (LIVE, FIGHT) have suggested potential harm, including increased heart rate, lack of improvement in left ventricular function, and possible worsening of outcomes.^33^, ^34^, ^35^, ^36^ Overall, while GLP‐1 RAs appear safe and beneficial in HFpEF with obesity, they should be used cautiously in patients with reduced LVEF until more definitive evidence becomes available.

#### Heart failure staging

**Figure 2:**
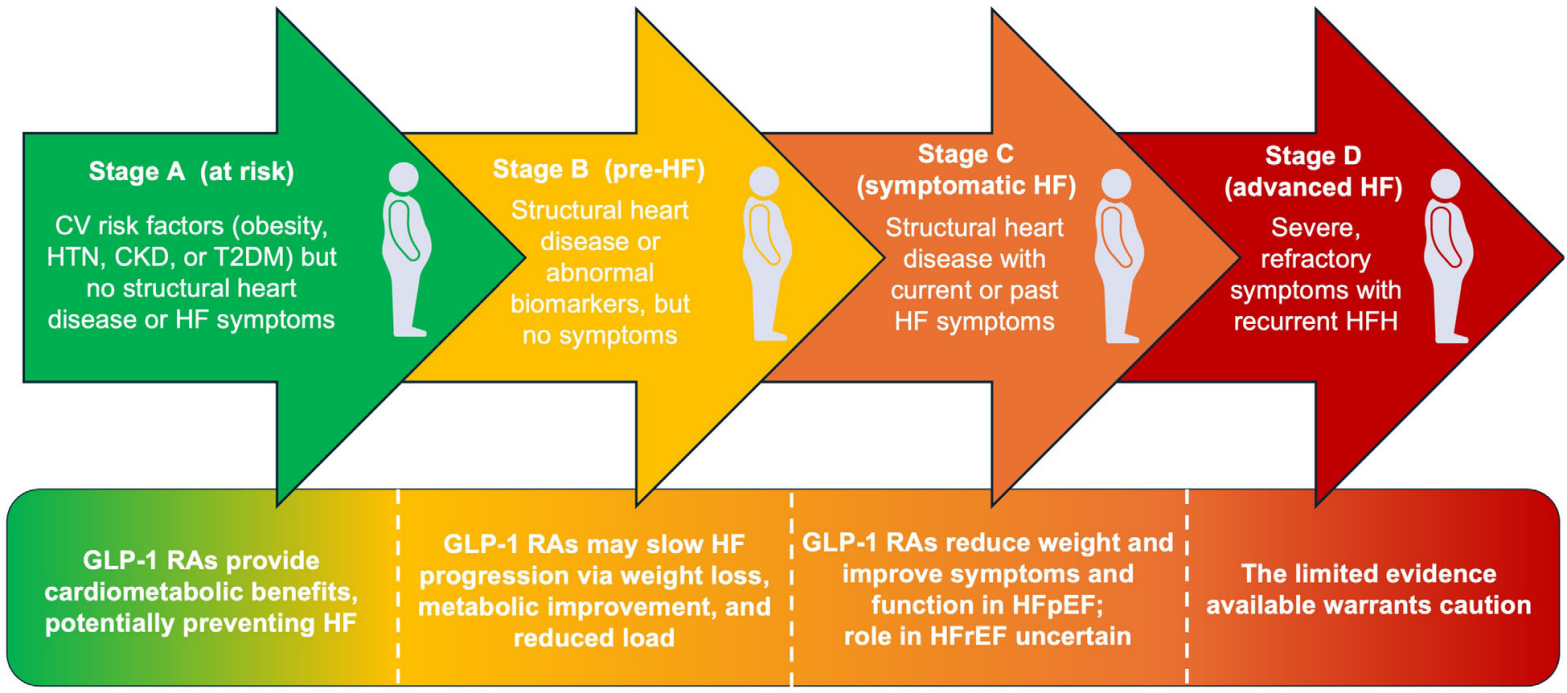
Role of glucagon‐like peptide‐1 receptor agonists (GLP‐1 RAs) across the Universal Definition of Heart Failure stages. CKD, chronic kidney disease; CV, cardiovascular; HF, heart failure; HFpEF, heart failure with preserved ejection fraction; HFrEF, heart failure with reduced ejection fraction; HFH, heart failure hospitalization; HTN, hypertension; T2DM, type 2 diabetes mellitus.

The HF stages (A–D) defined in the Universal Definition of HF^41^ offer a practical framework to guide the use of GLP‐1 RAs (*Figure* *2*):

- **Stage A (at risk)** – Patients with CV risk factors (obesity, hypertension, T2DM, chronic kidney disease) but without structural heart disease. This stage may represent an optimal window for GLP‐1 RA use, given their cardiometabolic benefits.^42^, ^43^
- **Stage B (pre‐HF) –** Patients with obesity and structural heart disease (i.e. coronary artery disease, left ventricular hypertrophy, elevated natriuretic peptides), but no HF symptoms. GLP‐1 RAs may help attenuate disease progression through weight loss, improved metabolic profile, and reduced haemodynamic load.^38^, ^44^, ^45^
- **Stage C (symptomatic HF)** – In patients with HFpEF and obesity, strong evidence supports GLP‐1 RA or GLP‐1 RA/GIP administration based on the STEP‐HFpEF and SUMMIT trials.^11^, ^12^, ^13^, ^46^ In HFrEF, the use of these drugs remains controversial, and an individualized approach with shared decision‐making is advisable given the limited and mixed evidence.^33^, ^34^, ^35^, ^36^, ^37^, ^38^
- **Stage D (advanced HF)** – Evidence supporting the use of GLP‐1 RAs in HFrEF is limited, as these patients have not been included in large‐scale randomized trials. As mentioned above, the FIGHT study, which enrolled patients with HFrEF following recent hospitalization and with NYHA class III–IV symptoms, raised concerns about a possible increase in HF hospitalizations in this population.^34^, ^35^ Further evidence is needed to establish the safety and efficacy of GLP‐1 RAs in these patients.

#### Other considerations for patient selection

In patients with T2DM but no HF, two large meta‐analyses of RCTs suggest that GLP‐1 RAs may reduce the risk of new‐onset HF and mortality.^47^, ^48^ This stage might represent a potentially favourable setting in which to consider GLP‐1 RAs. However, it should be noted that no dedicated large‐scale trials have yet been conducted to demonstrate a reduction in incident HF among patients with CV disease or at risk of HF.

Many patients with HF are poor bariatric surgery candidates due to advanced symptoms, CV risk, frailty, advanced age, or comorbidities, and they face a higher risk of perioperative complications compared with individuals without HF.^49^ In such cases, GLP‐1 RAs offer a pharmacological option to achieve meaningful and sustained weight loss. Moreover, in patients with suboptimal response or weight regain after bariatric surgery, subsequent use of GLP‐1 RAs has been shown to enhance weight loss.^50^, ^51^ However, HF‐specific benefits after surgery remain unproven and warrant dedicated prospective randomized trials.

### Dosing, tolerability, and safety considerations

The pharmacological implementation of GLP‐1 RAs in patients with obesity and HF requires careful and individualized consideration of dosing, tolerability, and safety.^52^ Given the unique physiological and logistical challenges associated with HF care, dosing strategies from trials in other populations may not be directly applicable. Notably, the target doses used in HF trials were the same as those employed in weight‐loss studies (i.e. subcutaneous semaglutide 2.4 mg weekly or tirzepatide up to 15 mg weekly) and were higher than the doses typically used in diabetes trials.^13^, ^46^, ^53^ Both the STEP‐HFpEF trial and the SUMMIT trial adopted gradual dose‐escalation protocols to mitigate side effects and early discontinuation.^11^, ^12^, ^13^ Despite these titration strategies, 13% of participants in STEP‐HFpEF and 6% in SUMMIT halted the treatment due to adverse events. Real‐world data from non‐HF obese patients have reported substantially higher discontinuation rates (up to 30–50%),^54^, ^55^ although these findings should be interpreted with caution given the potential for bias. In a recent comparative weight‐loss study comparing the two agents, overall discontinuation rates were similar, although gastrointestinal symptoms prompted withdrawal more frequently with semaglutide than with tirzepatide (6.6% vs. 2.7%).^56^ Unfortunately, no head‐to‐head comparison between the two molecules has been conducted in HF.

Real‐world experience suggests that some patients with HF may only tolerate lower doses, raising questions about whether submaximal regimens retain the same or, at least, meaningful cardiometabolic benefits as those observed in clinical trials. HFpEF trials have focused exclusively on subcutaneous administration. Oral semaglutide has demonstrated CV benefits only in high‐risk patients with diabetes in the SOUL (Semaglutide cardiOvascular oUtcomes triaL) trial,^57^ while a weight reduction effect in patients with overweight or obesity was shown in the OASIS 1 (Oral Semaglutide Treatment Effect in People with Obesity) trial (but at a dose more than three times higher than the target used in T2DM studies).^58^ More recently, a real‐world study reported that once‐daily oral semaglutide was associated with improvements in both health status and weight loss in HFpEF patients with T2DM and obesity.^59^ Oral formulations remain untested in patients with obesity and HFpEF, a gap of particular relevance given that many individuals with HF may be unfamiliar with injectable therapies, potentially complicating adherence.

While no major safety concerns have been reported in patients with impaired renal function, data remain lacking for those with end‐stage renal disease (estimated glomerular filtration rate [eGFR] <15 ml/min/1.73 m^2^), as this population was excluded from clinical trials. Another key limitation is the exclusion from outcome trials of patients with recent worsening HF, leaving clinicians uncertain about the safety of initiating GLP‐1 RA therapy during or shortly after acute decompensation. This exclusion is notable, as many therapies in HF have historically demonstrated the greatest benefit in recently hospitalized patients.^60^, ^61^ Finally, since patients with a history of pancreatitis were also excluded from major trials, caution is advised in this subgroup until further safety data become available (*Figure* *1*).

Some safety concerns have been raised regarding the use of GLP‐1 RAs in patients with HFrEF. In particular, these agents have been shown to increase heart rate over time,^33^, ^62^ thereby raising concerns about haemodynamic tolerance, potential symptom worsening, and ultimately increased risk of adverse outcomes.^63^ Small RCTs have further suggested potential harm of GLP1 RAs, mainly in patients with more advanced disease (NYHA class III–IV), possibly secondary to increased heart rate and heightened risks of arrhythmias and HF worsening.^34^, ^35^, ^36^ Moreover, preclinical studies suggest that GLP‐1RAs may alter myocardial substrate utilization from fatty acids toward glucose,^64^, ^65^ a metabolic switch that in the energetically compromised failing heart might further reduce efficiency.^65^ On the other hand, obesity itself is associated with an excess risk of adverse events in HFrEF, and in selected cases the benefits of weight reduction might offset these possible drawbacks.^28^, ^66^ Thus, in HFrEF patients where obesity appears to be a major contributor to symptoms, prescribing GLP‐1 RAs should be preceded by a discussion between the patient, the caregiver, and the clinician about the absence of supporting evidence.^38^ No clinical trial data are currently available for tirzepatide in HFrEF, and its use in this population remains unsupported.

### Practical considerations and side effect management in heart failure

As with other components of guideline‐directed medical therapy for HF, treatment with GLP‐1 RAs involves careful initiation, gradual dose escalation, and ongoing monitoring. However, tolerability remains a key concern, especially in HF populations who are often older, frail, and subject to complex polypharmacy.^67^ The relatively high incidence of side effects, meaningful discontinuation rates even in RCTs, and the inherent complexity of obesity‐related HFpEF underscore the importance of a multidisciplinary approach, including HF specialists, nurses, nutritionists, endocrinologists and rehabilitation experts, to optimize GLP‐1 RA or GLP‐1/GIP RA therapy and ensure adherence.

#### Gastrointestinal tolerability

As mentioned above, gastrointestinal side effects (nausea, vomiting, diarrhoea, and constipation) are the most frequently reported adverse events with GLP‐1 RAs. These symptoms typically emerge during dose escalation, are dose‐dependent and tend to resolve over time.^68^ However, in patients with HF, even mild anorexia or reduced oral intake may exacerbate hypovolaemia and hypotension.^69^ To mitigate gastrointestinal intolerance, therapy should begin at low (i.e. semaglutide at 0.25 mg/week, tirzepatide at 2.5 mg/week)^70^ or very low (i.e. semaglutide at 0.0675 mg/week)^71^ doses with gradual titration every 4 weeks to the maximum tolerated dose.

**Figure 3:**
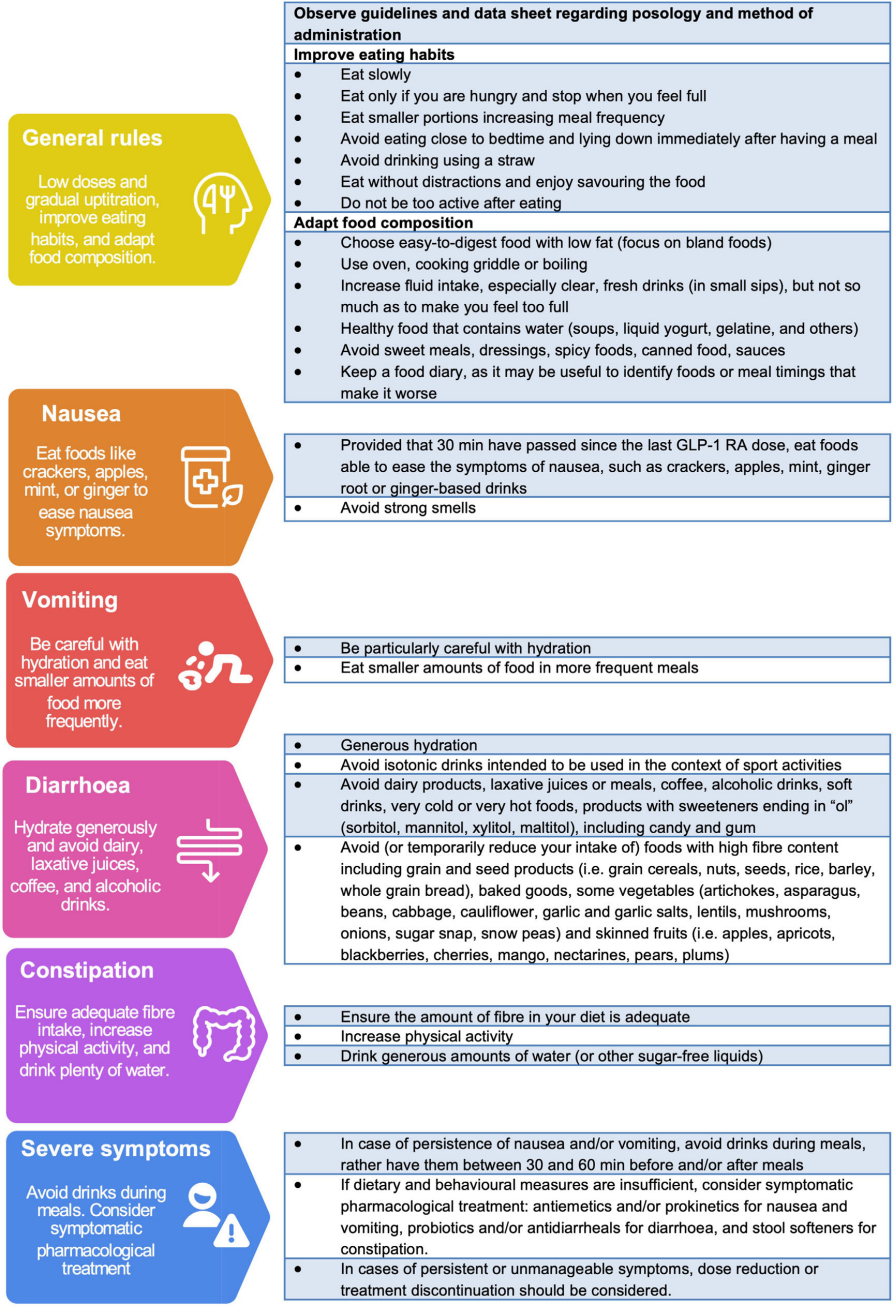
Management of glucagon‐like peptide‐1 receptor agonist (GLP‐1 RA)‐induced gastrointestinal side effects. Adapted from Gorgojo‐Martínez *et al*.^70^

Patient education plays a central role in improving adherence and minimizing discontinuation, particularly by setting realistic expectations and providing clear instructions on recognizing and managing side effects (*Figure* *3*).^70^ Behavioural and dietary modifications are key to improving tolerability. Patients should be encouraged to eat slowly, avoid meals when not hungry, stop eating at the early signs of fullness, and avoid lying down immediately after eating. Smaller, more frequent meals consisting of bland, easy‐to‐digest foods with low fat content are recommended, while high‐fat, spicy, canned, or highly sweetened foods should be avoided. Adequate hydration using small, frequent sips of clear fluids is essential, and the use of food diaries may help identify individual triggers. When symptoms occur, additional strategies include ginger or mint for nausea, cautious rehydration and dietary adjustments for vomiting or diarrhoea, and increased fibre, hydration, and mobility for constipation. In more persistent or severe cases, it is advisable to avoid drinking during meals, with fluids instead consumed 30 to 60 min before or after eating. Clinicians may also consider dose adjustments, temporary treatment interruptions, or symptomatic pharmacological management with agents such as domperidone. In all cases, a patient‐centred approach, combining slow titration, education, and proactive symptom management, is critical for successful long‐term use of GLP‐1 RAs in HF patients.^70^ Persistent or severe symptoms may necessitate dose reduction or discontinuation.^70^, ^72^

#### Volume depletion and hypotension

Glucagon‐like peptide‐1 receptor agonists may promote mild natriuresis and diuresis,^73^ contributing to blood pressure and body weight reduction.^22^ While these effects may benefit volume‐overloaded HF patients, potentially permitting diuretic dose reduction, they may also predispose to symptomatic hypotension or acute kidney injury, particularly in individuals on diuretics, renin–angiotensin–aldosterone system inhibitors, or with low baseline blood pressure.

Close monitoring of blood pressure, renal function, and volume status is strongly advised, especially during the first weeks of treatment to monitor for signs of hypovolaemia and guide timely therapeutic adjustments.^74^ In patients experiencing dizziness, orthostatic intolerance, or fatigue, temporary down‐titration of loop diuretics or antihypertensives may be warranted.^75^ Although major RCTs suggest that GLP‐1 RAs are generally very well tolerated from a haemodynamic standpoint, cautious use and individualized management remain essential in patients with advanced HF.

#### Nutritional status and risk of sarcopenia and cachexia

Glucagon‐like peptide‐1 receptor agonist treatment has been associated with reductions in lean mass in obese patients, a change that appears greater than that observed with other weight‐loss approaches and was also reported in the SUMMIT trial.^76^ The clinical relevance of this finding remains uncertain^77^ and warrants further investigations. While intentional weight loss may offer clinical benefits in patients with obesity‐related HFpEF, persistent anorexia and excessive weight loss in those with limited nutritional reserves may be detrimental, potentially leading to sarcopenia and HF‐related cachexia,^78^ a condition associated with poor outcomes.^79^

Regular assessment of body weight, nutritional intake, and body composition is therefore critical throughout treatment. In patients exhibiting excessive weight loss, signs of malnutrition, or declining functional status, early referral to a dietitian is advised. A concomitant programme of physical rehabilitation may be advisable to effectively counteract the loss of muscle mass associated with these therapies. In severe cases, the continuation of GLP‐1 RAs therapy should be re‐evaluated, and discontinuation may be warranted.

#### Pancreatic, intestinal and biliary tract complications

Glucagon‐like peptide‐1 receptor agonists may rarely be associated with acute pancreatitis, intestinal obstruction, and gallbladder disease, particularly in the setting of rapid weight loss.^80^, ^81^, ^82^ However, available data are mixed and not entirely consistent, and evidence in patients with HF is lacking. Patients should be educated to promptly report warning signs such as persistent abdominal pain, nausea, or vomiting. A prior history of pancreatitis is generally considered a relative contraindication to GLP‐1 RA therapy.

Routine imaging or laboratory screening for gastrointestinal complications is not warranted in asymptomatic individuals. However, any new or unexplained gastrointestinal symptoms should prompt rapid clinical assessment.

#### Other practical considerations

While earlier recommendations on the perioperative management of GLP‐1 RAs advised discontinuing treatment prior to surgery,^83^ more recent guidelines support continuing therapy in patients without significant gastrointestinal symptoms (such as severe nausea, vomiting, or inability to tolerate oral intake), with adjustments to fasting protocols to reduce the risk of gastric residue and aspiration (24 h for solids, 8 h for clear liquids containing ≥10% glucose, and 4 h for those with <10%).^84^ Conversely, in the presence of marked gastrointestinal symptoms, temporary discontinuation of GLP‐1 RAs should be considered (four to five drug half‐lives, the time required for its effect on gastric emptying to subside), with treatment resumption as soon as the patient can tolerate normal oral intake. Whenever feasible, regional anaesthesia should be preferred as the primary technique to further minimize aspiration risk in this population (*Table* *1*).^85^

**Table 1:** Key advice for perioperative management of patients taking glucagon‐like peptide‐1 receptor agonists

Continue GLP‐1 RAs perioperatively in patients without significant gastrointestinal symptoms (i.e. persistent vomiting, severe nausea, or inability to tolerate oral intake).
Fasting from solids for 24 h (clear liquids allowed) is advised before anaesthesia in patients on GLP‐1 RAs who do not have significant gastrointestinal symptoms.
Avoid high‐carbohydrate clear liquids (≥10% glucose) for 8 h before anaesthesia in patients on GLP‐1 RAs without significant gastrointestinal symptoms.
Allow only no‐ or low‐carbohydrate (<10% glucose) clear liquids (i.e. water, tea, black coffee) up to 4 h before anaesthesia in patients on GLP‐1 RAs without significant gastrointestinal symptoms.
For patients with significant gastrointestinal symptoms, it may be prudent to withhold GLP‐1 RAs before elective procedures requiring anaesthesia, although the optimal discontinuation period is not well established (withholding the drug for three to five half‐lives is generally considered sufficient to allow recovery of gastrointestinal motility and gastric emptying).
Prefer regional anaesthesia as the primary anaesthetic technique, when appropriate
After surgery, resume GLP‐1 RAs once the patient has returned to regular oral intake and is tolerating food without symptoms.

GLP‐1 RA, glucagon‐like peptide‐1 receptor agonist.

Primarily adapted from Oprea *et al*.^84^; additional elements informed by El‐Boghdadly *et al*.^85^

Patients and physicians should be aware that discontinuing GLP‐1 RA therapy may lead to weight regain, which has been observed to consist disproportionately of fat rather than lean mass, potentially exacerbating sarcopenic obesity and worsening outcomes in HF patients.^86^ Maintaining regular physical activity may help mitigate this rebound.^87^

In summary, while GLP‐1 RAs offer a promising therapeutic option in patients with HFpEF and obesity, their side effect profile requires proactive and individualized management. Optimizing tolerability and safety hinges on careful dose escalation, close monitoring of fluid and nutritional status, and thorough patient education.

## Potential synergies with heart failure drugs

The evolving therapeutic landscape of HF, particularly in patients with cardiometabolic comorbidities such as obesity and T2DM, has introduced new opportunities for combination strategies targeting distinct yet complementary pathophysiological pathways.^2^ Indeed, the pleiotropic effects of GLP‐1 RAs may enhance clinical benefits in HF patients through multiple mechanisms.^31^, ^88^, ^89^, ^90^

### Sodium–glucose co‐transporter 2 inhibitors

Sodium–glucose co‐transporter 2 inhibitors share some metabolic and haemodynamic benefits with GLP‐1 RAs, including diuresis and improved myocardial energetics.^9^, ^91^ Importantly, their mechanisms may be complementary: SGLT2 inhibitors have been shown some effect in reducing plasma volume by promoting glucosuria and natriuresis, while GLP‐1 RAs target insulin resistance, systemic inflammation, and visceral adiposity.^92^, ^93^ Early data suggest potential additive effects on weight, glycaemic control, blood pressure, and potentially vascular stiffness,^94^, ^95^ supporting their combined use, particularly in obesity‐related HFpEF, where both volume overload and metabolic dysregulation contribute to disease progression. In patients with T2DM, co‐administration with SGLT2 inhibitors may offer additional outcome benefits and should be preferred.^96^

### Angiotensin receptor–neprilysin inhibitors

Sacubitril/valsartan improves neurohormonal modulation and promotes cardiac remodelling.^97^, ^98^ By inhibiting neprilysin, it may also slow the degradation of endogenous GLP‐1 and glucagon, thereby enhancing the activity of GLP‐1 and other peptides, and providing a pharmacodynamic rationale for potential synergy or interaction with GLP‐1 RAs.^99^, ^100^ Although clinical trial data on this combination are lacking, such interactions may amplify the benefits on cardiac structure and function, particularly in patients with HF and features of the metabolic syndrome. While concerns about additive hypotension or gastrointestinal side effects remains theoretical, close clinical monitoring is recommended. It should also be noted that, while sacubitril/valsartan has proven efficacy in patients with HFrEF, the use of GLP‐1 RAs in this setting should be approached with caution (see above).

### Mineralocorticoid receptor antagonists

Mineralocorticoid receptor antagonists reduce myocardial fibrosis, sodium retention, and inflammation – pathways that potentially intersect with the anti‐inflammatory and anti‐fibrotic actions of GLP‐1 RAs.^101^, ^102^ Combination therapy may thus yield complementary effects on myocardial remodelling and systemic metabolic inflammation, which are key features of cardiometabolic HFpEF and HFrEF. While definitive outcome data on these combinations remain limited, mechanistic plausibility and early exploratory findings justify further investigation. Future trials should explore tailored polypharmacy approaches targeting HF phenotypes enriched for metabolic dysfunction, with the goal of optimizing clinical outcomes while mitigating adverse effects and improving patient stratification.

## Knowledge gaps and future directions

**Figure 4:**
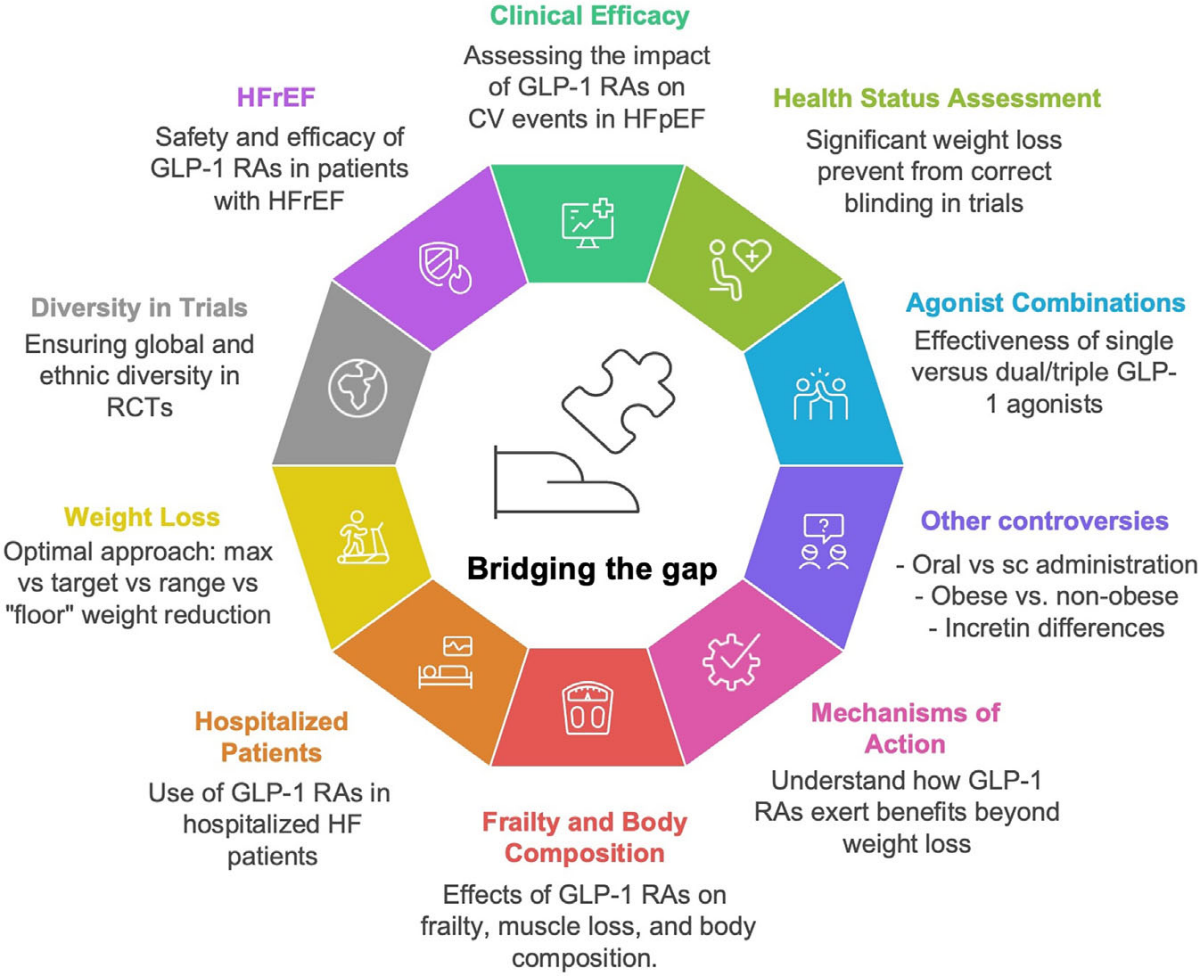
Knowledge gaps on the use of glucagon‐like peptide‐1 receptor agonists (GLP‐1 RAs) in patients with heart failure and obesity. CV, cardiovascular; HF, heart failure; HFpEF, heart failure with preserved ejection fraction; HFrEF, heart failure with reduced ejection fraction; RCT, randomized controlled trial; sc, subcutaneous.

Several key questions remain unanswered regarding the use of GLP‐1 RAs in patients with HF and obesity, requiring further investigation across clinical, mechanistic, and implementation domains (*Figure* *4*).

### Effect of GLP‐1 RAs on cardiovascular outcomes in HFpEF

In prior CV outcome trials in patients with T2DM but without HF, GLP‐1 RAs reduced the risk of myocardial infarction, stroke and CV death. It is conceivable that similar benefits might extend to HFpEF populations, although the absolute event rates for such outcomes are typically lower. Indeed, participants in the STEP‐HFpEF and SUMMIT trials were at relatively low risk, as evidenced by the limited number of HF events (38 and 55 hospitalizations, respectively),^11^, ^13^ compared with much higher event rates in trials of SGLT2 inhibitors and finerenone (i.e. DELIVER: 1872; FINEARTS‐HF: 1866).^103^, ^104^ This reflects a broader challenge in HFpEF research, where non‐CV (often non‐modifiable) causes of death predominate, limiting the ability to demonstrate CV mortality benefits.^105^ To establish the efficacy and safety of GLP‐1 RAs in HFpEF, larger trials enrolling higher‐risk populations and potentially incorporating broader endpoints, such as 5‐point major adverse cardiac events, kidney function, sleep apnoea, and cognitive health, are needed to fully characterize their therapeutic potential. Currently, the only large‐scale outcome trial underway is MARITIME‐HF (NCT07037459), which will randomize 5056 patients with obesity and HFpEF to receive once‐monthly subcutaneous maridebart cafraglutide (known as MariTide; a GLP‐1 RA with GIP antagonism) or placebo, with the primary endpoint of time to first CV death or HF event.

### Lack of safety and efficacy evidence in patients with HFrEF

As discussed above, the evidence for GLP‐1 RAs, dual GLP‐1/GIP RAs, and other incretin‐based therapies in HFrEF remains limited and inconclusive, with concerns about potential adverse effects such as arrhythmias and worsening HF. Dedicated trials in this high‐risk population are warranted.

### GLP‐1 RA alone or GLP‐1/GIP RA, or alternative weight loss therapies in HFpEF

A key unanswered question is whether GLP‐1 receptor agonism is sufficient to improve outcomes in HFpEF, or whether additional benefits can be achieved by combining it with other incretin‐based pharmacologic actions. With over 150 weight‐loss drugs currently in development, the therapeutic added value of dual (i.e. GLP‐1/GIP RAs) or triple agonists (i.e. retatrutide with glucagon receptor agonism) is under active investigation.^106^ Understanding how these combinations differentially affect HF and broader CV endpoints will be essential for guiding future therapeutic strategies.

### GLP‐1 RAs and frailty in HFpEF

As also mentioned above, treatment with GLP‐1 RAs may result in loss of both adipose tissue and skeletal muscle, potentially exacerbating frailty in patients with HF, a population already prone to muscle wasting and cardiac cachexia.^78^, ^107^. However, in STEP HFpEF and SUMMIT trials, patients showed improved health status and greater walking distance, suggesting reduced frailty.^11^, ^12^, ^13^, ^46^ The relative loss of adipose tissue versus skeletal muscle mass remains unclear, as body composition studies in HFpEF are lacking. It is plausible that fat loss predominates over skeletal muscle, preserving or even enhancing functional capacity. Dedicated mechanistic trials are needed to assess the effects of GLP‐1 RAs on body composition and clarify their role in patients vulnerable to sarcopenia.

### GLP‐1 RAs in hospitalized HFpEF patients – benefits and adverse events?

Hospitalization offers a key window to initiate HF therapies under specialist care. While most guideline‐directed medical therapies were first validated in outpatients and later in smaller hospitalized cohorts, the safety of GLP‐1 RAs in recently hospitalized HF patients remains uncertain. Gastrointestinal side effects, particularly during the early treatment phase, may disrupt fluid balance and complicate recovery during or shortly after discharge. Future studies should include hospitalized patients, either within large trials or as dedicated cohorts, to properly assess the safety and efficacy of GLP‐1 RAs in this vulnerable HFpEF population.

### Optimal GLP‐1 RA‐induced weight loss in patients with obesity and HFpEF

In patients with obesity and HFpEF receiving GLP‐1 RAs, the optimal weight‐loss strategy remains uncertain, as does the extent to which potential benefits may occur independently of weight loss. Potential approaches include pursuing maximal weight reduction, targeting a specific absolute weight, maintaining weight within a defined range, or ceasing weight loss upon reaching a predefined ‘floor’ weight to prevent excessive loss. These strategies need to be tested in prospective trials.

### Trials of GLP‐1 RAs in HFpEF in different parts of the world and different ethnicities

Both HFpEF phenotype and body composition vary substantially across regions, countries and ethnicities. Trials conducted in diverse geographic and ethnic populations are therefore necessary to ensure the generalizability of findings.^108^

### Other controversies and open questions in the utilization of GLP‐1 RAs in both HFpEF and HFrEF

Despite promising preliminary data, critical knowledge gaps remain regarding the use of GLP‐1 RAs in HF. Future trials should evaluate hard clinical outcomes across diverse settings, compare the efficacy of oral versus subcutaneous formulations, assess the impact of lifestyle interventions and optimal weight loss strategies, explore differences in response between obese and non‐obese patients and across incretin‐based therapies, while further elucidating underlying mechanisms of action (*Table* *2*).

**Table 2:** Possible future trials investigating glucagon‐like peptide‐1 receptor agonists in heart failure with preserved and reduced ejection fraction

Oral vs. subcutaneous GLP‐1 RAs in HFpEF and HFrEF
Communication methods with patients/carers in HF and obesity
Optimal diet and exercise with GLP‐1 RAs
RCT in HFrEF with obesity – clinical endpoints
RCT in HFpEF and HFrEF in non‐obese – clinical endpoints
RCT in hospitalized obese HFpEF and HFrEF – clinical endpoints
Head‐to‐head trials: GLP‐1 RAs vs. novel weight loss drugs
Mechanistic trials in HFpEF and HFrEF – GLP‐1 RA effects
Trials on optimal weight loss in HFpEF and HFrEF

GLP‐1 RA, glucagon‐like peptide‐1 receptor agonist; HF, heart failure; HFpEF, heart failure with preserved ejection fraction; HFrEF, heart failure with reduced ejection fraction; RCT, randomized controlled trial.

## Conclusions

Glucagon‐like peptide‐1 receptor agonists have emerged as a promising therapeutic option for patients with obesity and HFpEF. Recent trials have demonstrated benefits in terms of weight loss, exercise capacity, and quality of life, though their impact on hard CV outcomes, especially HF hospitalization and mortality, remains uncertain. Definitive outcome trials in HFpEF are underway. In contrast, their role in HFrEF is less well defined, with available evidence limited to small trials with few events and no confirmation from large‐scale outcome trials.

This expert consensus provides an updated, evidence‐based framework for the pragmatic integration of GLP‐1 RAs into HF care, highlighting both established evidence and remaining knowledge gaps relevant to everyday clinical practice. Looking ahead, adequately powered clinical and mechanistic trials remain essential to address the many unresolved questions surrounding their use in this population.

### Funding

G.G.S. is supported by research grants from the DZHK (German Centre for Cardiovascular Research – 81X3100210; 81X2100282); the Deutsche Forschungsgemeinschaft (DFG, German Research Foundation – SFB‐1470–A02); the European Research Council – ERC StG 101078307 and HI‐TAC (Helmholtz Institute for Translational AngioCardioScience).

**Conflict of interest**: L.M. has received a travel grant from Boehringer Ingelheim. G.S. reports grants and personal fees from CSL Vifor, Boehringer Ingelheim, AstraZeneca, Servier, Novartis, Cytokinetics, Pharmacosmos, Medtronic, Bayer, and personal fees from Roche, Abbott, Edwards Lifescience, TEVA, Menarini, INTAS, GETZ, Hikma, Laboratori Guidotti, and grants from Boston Scientific, Merck. W.M. reports research grants from Novartis, Vifor, Medtronic, Biotronik, Abbott and Boston Scientific. A.A. reports speaker honoraria from Boston Scientific, Medtronic and Bayer. B.B. reports consultation or advisory roles for Abiomed/Johnson and Johnson, Bayer, Boehringer Ingelheim, Cardurion, Cytokinetics, Eli Lilly, Medtronic, Merck, Idorsia, Novo Nordisk, Regeneron, Renovacor, Roche, Salubris, Sanofi‐Aventis, scPharmaceuticals, Vifor, Respicardia/Zoll. O.C. has received grants from Servier. R.M.I. participated in speaking engagements with AstraZeneca, Bayer, Novo Nordisk, Boehringer Ingelheim, Novartis, Daiichi Sankyo, Bruno Pharma, Sanofi; serves on advisory boards with AstraZeneca and Novo Nordisk; has received non‐industry fees from PACE‐CME. M C.P. has received research funding from the British Heart Foundation, the UK National Institute for Health and Care Research, Boehringer Ingelheim, Roche, SQ Innovation, AstraZeneca, Novartis, Novo Nordisk, Medtronic, Boston Scientific, and Pharmacosmos; consulting fees for clinical trial committees or consulting from Abbott, Akero Therapeutics, Applied Therapeutics, Amgen, AnaCardio, Biosensors International, Boehringer Ingelheim, Corteria, Novartis, AstraZeneca, Novo Nordisk, AbbVie, Bayer, Horizon Therapeutics, Foundry, Takeda, Cardiorentis, Pharmacosmos, Siemens, Eli Lilly, Vifor, NewAmsterdam Pharma, Moderna, Teikoku Pharma, LIB Therapeutics, 3R Lifesciences, Reprieve, FIRE 1, Corvia Medical, and Regeneron; speaking honoraria from Boehringer Ingelheim, Novartis, AstraZeneca, Novo Nordisk, Pharmacosmos, Eli Lilly, and Vifor; travel support from Boehringer Ingelheim, Novartis, AstraZeneca, Novo Nordisk, Pharmacosmos, Eli Lilly, and Vifor; has participated on data safety monitoring boards for AstraZeneca, Teikoku, and Moderna; and serves as the Director of Global Clinical Trials Partners. G.G.S. report grant from Novo Nordisk and personal fees from Boehringer Ingelheim and Pfizer. D.S. received speaker and/or advisory board fees from Boehringer Ingelheim, Janssen, MSD, Novo Nordisk, Novartis, AstraZeneca, Dompè, Viatris and Gossamer Bio. M.M. reports consulting honoraria of small amounts from AstraZeneca, Bayer, Boehringer Ingelheim, Novo Nordisk and Roche Diagnostics in the last 3 years. N.G. reports honoraria from AstraZeneca, Bayer, Boehringer Ingelheim, Lilly, Novartis, Novo Nordisk, Roche Diagnostics, NP Medical and Echosens. All other authors have nothing to disclose.
